# Polysaccharides from *Artemisia argyi* leaves: Environmentally friendly ultrasound-assisted extraction and antifatigue activities

**DOI:** 10.1016/j.ultsonch.2024.106932

**Published:** 2024-05-31

**Authors:** Jiahui Yu, Ying Cao, Feng He, Fu Xiang, Shuzhen Wang, Wenbing Ke, Wei Wu

**Affiliations:** a Hubei Key Laboratory of Economic Forest Germplasm Improvement and Resources Comprehensive Utilization, Hubei Collaborative Innovation Center for the Characteristic Resources Exploitation of Dabie Mountains, Huanggang Normal University, Huangzhou 438000, China; b Department of Infectious Diseases, Huangshi Hospital of Traditional Chinese Medicine, Huangshi 435000, China

**Keywords:** Polysaccharide, *Artemisia argyi* leaves, Extraction, ultrasonic, Antifatigue

## Abstract

*Artemisia argyi* leaf polysaccharide (AALPs) were prepared through ultrasound-assisted extraction (UAE), and their antifatigue activities were evaluated. Extraction was optimized using response surface methodology (RSM), which yielded the following optimal UAE conditions: ultrasonication power of 300 W, extraction temperature of 51 °C, liquid:solid ratio of 20 mL/g, and ultrasonication time of 47 mins. The above optimal conditions resulted in the maximum extraction rate of 10.49 %. Compared with hot water extraction (HWE), UAE supported higher yields and total sugar, uronic acid, and sulfate contents of AALPs. Meanwhile, AALP prepared through UAE (AALP-U) exhibited higher stability due to its smaller particle size and higher absolute value of zeta potential than AALP prepared through HWE (AALP-H). In addition, AALP-U demonstrated stronger antioxidant activity than AALP-H. In forced swimming tests on mice, AALP-U could significantly prolong swimming time with a dose-dependent effect, increase liver and muscle glycogen levels, and improve other biochemical indices, thus showing great potential for application in functional food.

## Highlights

• Ultrasound-assisted extraction of *Artemisia argyi* leaf polysaccharide (AALP) was optimized by RSM.
• Different extraction methods have a considerable influence on chemical composition, monosaccharide composition, and molecular weight of AALPs.
• AALP prepared through UAE (AALP-U) and HWE (AALP-H) all exhibited remarkable antioxidant activities.
• AALP-U exhibited considerable antifatigue activity and has excellent application prospects in the field of food and medicine.

## 1 Introduction

Mugwort (*Artemisia argyi* Levl. et Vant.) is Compositae herbaceous plant of the genus *Artemisia*. It is widespread in Asia, Europe, and North America as well as in cool temperate and subtropical regions. *A. argyi* leaf (AAL), the mature leaf of mugwort, is a traditional medicinal plant resource in China. It has the functions of clearing heat and resolving phlegm, warming channels to stop bleeding, and regulating intestinal immunity [1]. The main chemical components of AAL include polysaccharides, phenolic acids, flavonoids, essential oils and terpenoids [2], [3], [4], [5], [6]. In recent years, its application range has broadened with the increase in the research on its chemical composition and pharmacology. However, its application in China mainly focuses on primary processing, such as processing for use in moxibustion and instant food. Mugwort powder, a by-product of moxibustion, is discarded as a nonmedicinal component. This situation results in serious resource waste and environmental pollution. AAL is abundant in polysaccharides, a type of glycoprotein with various biological activities. They have various pharmacological actions, such as antioxidant, immunomodulatory, and antitumor actions, which are closely related to *A. argyi* leaf polysaccharides (AALPs) [[2], [7], [8]]. Therefore, extracting AALPs can not only help extend the application scope of AAL and provide products with high additional value, it can also reduce the environmental pollution caused by waste disposal and promote the diversified development of the *A. argyi* industry.

With rapid economic development and increasingly fierce social competition, a growing number of people are bearing the overload of work and massive pressure of life. Fatigue is a physical state that reduces the movement ability of the human body, which could force people to stop exercising or decrease the intensity of exercise to avoid injury [9]. Fatigue not only reduces the ability for physical exercise, it also leads to a decline in human immunity and muscle contraction function as well as causes nervous central system disorders. Long-term fatigue exerts a direct effect on human health [10]. Therefore, mining natural ingredients with strong antifatigue activity and developing functional food to relieve fatigue is of profound importance. Compared with sildenafil and other antifatigue drugs, polysaccharides from natural sources are safer and more effective and have gradually become the focus of the research on antifatigue functional food.

The most common extraction methods for AALP include hydrothermal, acid, and alkali extraction methods. Traditional hydrothermal extraction methods have low extraction efficiency and polysaccharide yields, and acid and alkali extraction methods have high requirements for experimental equipment and easily cause changes in polysaccharide structure. Ultrasonic-assisted extraction (UAE) could shorten extraction times and has wide adaptability and low operating costs. It results in low invalid impurities in the extracted solution, and the extracted active ingredients are easy to refine. Its core principle is to accelerate the movement of solute molecules caused by medium propagation in cells, resulting in plant cell wall rupture to release cell contents for the extraction of biological active ingredients, thereby improving yield rates. Lu *et al.* [11] and Wang *et al*. [12] found that compared with those obtained through other extraction methods (hot water extraction [HWE], pressure-assisted extraction, and high-pressure homogeneous extraction), polysaccharides prepared through UAE showed stronger activity in the removal of hydroxyl radicals, 1,1-diphenyl-2-picrylhydrazyl (DPPH) and 2,2ʹ-azino-bis (3-ethylbenzothiazoline-6-sulfonic acid) ammonium salt (ABTS). Moreover, UAE has no effect on the chemical structure of polysaccharides, which all retained their classic structures.

In consideration of the above, this research aims to investigate the influence of UAE parameters on the yield of AALP, optimize AALP extraction parameters by using response surface methodology (RSM), and characterize the physical and chemical properties of polysaccharides derived through different extraction methods. At the same time, the antioxidant activity in vitro and antifatigue activity in vivo of AALPs were measured, and the antifatigue mechanism of AALPs was preliminarily explored. This work will provide a theoretical basis for the application of AALPs in food, medicine, and other fields as well as reliable technical support and a reference method for related research on other plant polysaccharides.

## 2 Materials and methods

### 2.1 Materials and reagents

AAL was acquired from Hubei Lishizhen Biotechnology Co. It was dried at 60 °C, sieved to 60 mesh and stored in a drying oven. All chemical reagents used in the experiment were of analytical purity, and supplied by Sinopharm Chemical Reagent Co., Ltd. Distilled water was used throughout the experiment.

### 2.2 UAE of AALP

UAE was performed in reference to the method of Ying et al. [13], as follows: A total of 1.000 g of AAL powder was weighed, mixed with purified water in proportion, and placed in an XH-2008 D ultrasonication extractor (Xianghu Technology, Beijing, China). This process was repeated twice under the set conditions of extraction temperature, ultrasonication time, liquid:solid ratio, and ultrasonication power. The extracts were combined and subjected to protein removal through the Sevage method, and then was adjusted to pH 4.0, activated charcoal (2 g/100 ml) was added, shaken well and incubated at 50 °C for 10 mins. The extract was concentrated to a volume of 50 mL and then mixed with ethanol to achieve a final concentration of 80 % (v/v). The mixture was stored overnight at 4 °C, and the resulting precipitates were recovered by centrifugation at 4500 rpm for 15 mins. The crude polysaccharide was then freeze-dried for use. The levels of the single-factor test for investigating the effects of temperature, ultrasonication power, liquid:solid ratio, and extraction time on the AALP extraction rate were arranged as follows: extraction temperature (*X_1_*: 30 °C, 40 °C, 50 °C, 60 °C, and 70 °C), ultrasonication power (*X_2_*: 150, 200, 250, 300, and 350 W), liquid:solid ratio (*X_3_*: 10, 15, 20, 25, and 30 mL/g) and extraction time (*X_4_*: 15, 30, 45, 60, and 75 mins).

The determination of polysaccharide content was carried out using the phenol–sulfuric acid method. A total of 10 mg of glucose, which was dried to a constant weight at 95 °C, was accurately weighed in a 250 mL volumetric bottle and supplemented with water by the scale. A total of 0.1, 0.2, 0.3, 0.4, 0.5, 0.6, 0.7, and 0.8 mL of standard glucose solution were collected, added with water to the total volume of 1.0 mL, mixed with 0.5 mL of 6 % phenol and 2.5 mL of concentrated sulfuric acid, kept at room temperature for 20 mins. Absorbance values at 490 nm were then measured. In the standard curve, the concentration of glucose solution (mg/L) is the horizontal coordinate, and the value of optical density is the vertical coordinate. A total of 5 mL of AALP solution was collected and mixed with 0.5 mL of distilled water then measured in accordance with the above procedure. The yield of AALP was expressed as Y = C/W × 100 %, where C is the crude AALP content (g), and W is the mass of AAL powder (g).

### 2.3 HWE of AALP

We performed HWE following the method of He *et al.* [14] with appropriate improvements. After pretreatment, AAL powder was mixed with distilled water as the extraction solvent. Extraction was performed three times at 90 °C with a liquid:solid ratio of 25 (mL/g), and all the extraction liquid was collected. The remaining processing steps and yield calculation in HWE were the same as those in UAE.

### 2.4 Optimization of AALP extraction conditions by RSM

The optimization of AALP extraction was carried out using RSM. Extraction temperature (A), ultrasonication power (B), liquid:solid ratio (C), and extraction time (D) were selected as the response surface design factors. The levels of each factor were determined in accordance with the single-factor experimental results. The AALP yield was regarded as the response value in accordance with the Box–Behnken principle. The process conditions were optimized using the response surface test, which involved four factors and three levels. The test data was analyzed and the predicted response value was calculated using Design Expert software (Version 13.0, Stat-Ease, Inc., Minneapolis, MN).

### 2.5 Characterization of AALP

#### 2.5.1 Chemical composition analysis

The neutral sugar contents in AALP prepared through UAE (AALP-U) and AALP prepared through HWE (AALP-H) were determined using the phenol–sulfuric acid method described in 2.2. The glucuronic acid (GluA) content in AALPs was determined by using the carbazole sulfate colorimetric method with GluA as the standard [15], and the total sugar content was represented by the sum of neutral sugar and GluA contents. BCA kits were utilized to determine the total protein content in the sample with bovine serum protein as the standard [16]. The sulfate content of AALPs was determined in accordance with the method of Tabataba [17].

#### 2.5.2 Determination of the monosaccharide composition of AALPs

The monosaccharide composition of AALPs was analyzed through HPLC and PMP derivatization in accordance with the report of Zou [18]. Briefly, approximately 50 mg of AALP were hydrolyzed in a calibration tube with 5 ml 2 mol/L trifluoroacetic acid (TFA) at 110 °C for 6 h and dried with nitrogen. The residual TFA was removed with methanol washing and drying for 3 times. The hydrolysate was re-dissolved in ultrapure water and derived with 0.3 mol/L NaOH and 0.5 mol/L PMP ((1-phenyl-3-methyl-5-pyrazolone)-methanol in a 70 °C water bath for 60 mins, cooled, and was neutralized with 0.3 mol/L HCl, then extracted with chloroform until organic layer was colorless. The supernatant was filtered through a 0.22 μm microporous membrane, and collected the sample derivatives for test. Meanwhile, the monosaccharide standards (mannose, rhamnose, gluconic acid, galacturonic acid, galactose, arabinose, fucose, and glucose) were treated with same method. All samples were analyzed on an Agilent 1,260 HPLC system equipped with C_18_ column (4.6 mm × 250 mm, 5 μm), with the following settings: eluting solvent: phosphate buffer (pH 6.8) − acetonitrile (18: 82, v/v); flow rate 0.8 mL/min; column temperature 30 °C; detection wavelength 254 nm; injection volume: 10 μL.

#### 2.5.3 Determination of molecular weight

The molecular weight (Mw) distribution of AALP-H and AALP-U samples was determined by high performance liquid phase gel permeation chromatography (HPGPC) [19]. The HPGPC conditions were as follows: Agilent 1260 HPLC equipped with TSK-GelG4000SWXL column (7.8 mm × 300 mm) and RID detector, flow rate 0.7 mL/min, mobile phase: 0.1 mol/L NaCl, injection volume 10 μL, column temperature 35 °C. A standard curve of molecular weight distribution was constructed using dextran of different molecular weights, and the molecular weight distribution (Mw) of each sample was calculated.

#### 2.5.4 Infrared spectroscopy

A total of 2 mg of AALP samples and KBr powder were ground evenly, pressed into disks, and scanned through Fourier transform infrared spectroscopy (FT-IR) in the 400–4000 cm^−1^ range. The infrared absorption spectra of polysaccharide samples were thus obtained.

#### 2.5.5 Zeta potential and particle size measurement

AALP-H and AALP-U solutions (2.0 mg/mL) were prepared, and their zeta potential value and particle size were measured by using a Marvin NanoZS90 nanometer and zeta potential analyzer at room temperature [20].

### 2.6 Determination of antioxidant activity in vitro

AALP and ascorbic acid solutions (positive control) were prepared with different concentrations of 0.2, 0.4, 0.6, 0.8, 1.0, 2.0, 3.0, 4.0, and 5.0 mg/mL to assess antioxidant activity in vitro. The main evaluation indicators, including DPPH, ABTS, and hydroxyl radical scavenging activities, were determined in accordance with previous reports [21], [22], [23].

### 2.7 Antifatigue activity of AALPs

**Fig. 1:**
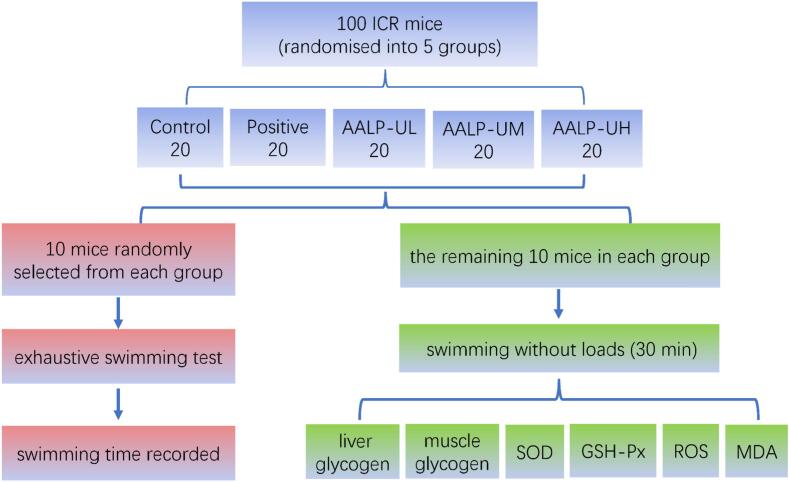
A flowchart of the anti-fatigue activity evaluation test.

Fig. 1 presents a flowchart of the anti-fatigue activity evaluation test.

#### 2.7.1 Design of the animal experiment scheme

For our animal experiments, Beijing Vital River Laboratory Animal Technology Co., Ltd supplied healthy SPF-grade male ICR mice, 6–8 weeks old, 18–22 g. In all, 100 mice were randomised into five groups (the test period was set at 30 days.), and the dosage design was as shown in Table 1. The body weight of mice was measured on days 0, 6, 12, 18, 24, and 30, and the growth of mice was observed. All animal experiments were conducted in accordance with the National Institutes of Health Guide for Care and Use of Laboratory Animals, and were approved by Huanggang Normal University Animal Ethics Committee (20230801).

**Table 1:** Animal experimental groups.

Group	Administration	Dose	N
Control	Normal saline	*	20
Positive	Ginsenoside	50 mg/kg/d	20
AALP-UL	AALP-U	50 mg/kg/d	20
AALP-UM	AALP-U	100 mg/kg/d	20
AALP-UH	AALP-U	200 mg/kg/d	20

Note: *: The volume of normal saline administered to the control group was identical to the volume of reagent administered to the other groups.

#### 2.7.2 Exhaustive swimming test

The operational procedure in our test was improved with slight modifications on the basis of the reported literature [24]. After 30 days, 10 mice were randomly selected, affixed with a tin wire (5 % of body weight) on the tail, and placed in a 35 cm deep swimming pool (100 cm × 100 cm × 80 cm) containing water with a temperature of 30 °C ± 1 °C. The time from beginning of the test to exhaustion was recorded.

#### 2.7.3 Determination of liver glycogen, muscle glycogen, superoxide dismutase, glutathione peroxidase, ROS, and MDA levels in mice

The biochemical parameters of mice were analyzed according to the method reported by Qi *et al*. [25]. Another 10 mice in each group were forced to swim without weight for 30 mins. Blood samples were drawn from the orbit immediately after the test ended; allowed to clot for 15 mins at room temperature; and centrifuged at 3200 rpm for 8 mins at 4 °C to collect serum, which was refrigerated for later use. The levels of superoxide dismutase (SOD), glutathione peroxidase (GSH-Px), reactive oxygen species (ROS), and MDA in serum were determined in accordance with the procedures included with kits. At the same time, liver and hind leg muscles were collected, rinsed with normal saline, and dried with filter paper. A total of 100 mg of liver and 500 mg of muscle were added to tubes. SOD, GSH-Px, ROS, and MDA levels in serum were measured in accordance with the instructions included with kits, and the contents of liver and muscle glycogen were analyzed at the same time.

### 2.8 Data statistics and analysis

Design Expert was applied to design the response surface optimization experiment of AALP extraction, analyze data, and draw response surface diagrams. SPSS 23.0 software was employed for multiple comparison through one-way ANOVA, and *p* < 0.05 presented a significant difference. Microsoft Excel 2016 was used for data processing, and Origin 2021 was utilized for charting. All experiments were repeated three times and data expressed as mean ± SD.

## 3 Results and analysis

### 3.1 UAE of AALP

#### 3.1.1 Single-factor test

**Fig. 2:**
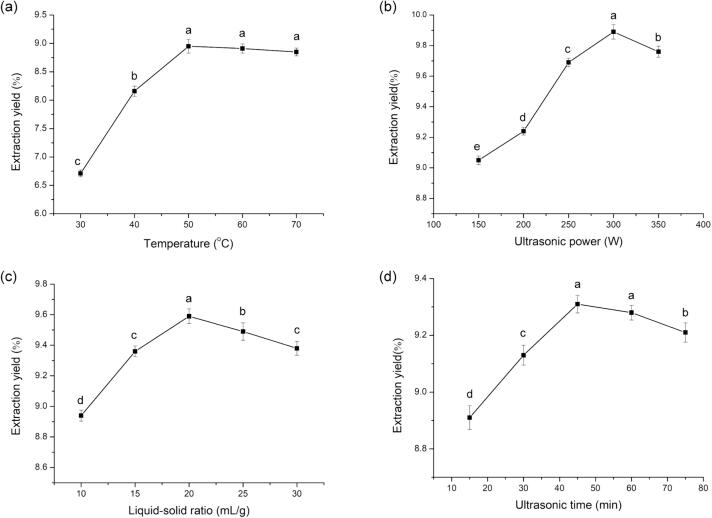
Effects of temperature (a), ultrasonic power (b), liquor to solid ratio (c), ultrasonic time (d) on AALP yield. The different letters represent significant differences (*p* < 0.05).

Fig. 2 (A) depicts that the AALP extraction rate showed a clear upward trend with increasing extraction temperature. When the extraction temperature reached 50 °C, the yield rate peaked and then slowly declined; this trend was similar to that shown by the yield of agaric polysaccharide obtained through UAE with temperature [26]. Although increasing temperatures help improve the solubility and mass transfer efficiency of polysaccharides, cavitation buffering due to the increase in vapor pressure at high temperature weakens ultrasonication cavitation [27], [28]. Therefore, extraction temperatures of 40 °C, 50 °C, and 60 °C were selected for subsequent tests.

Ultrasonication power is an important parameter in acoustic cavitation and closely related to cavitation bubble number, collapse energy, and cavitation [29]. Fig. 2(B) shows that AALP yield increased with the increase in ultrasonication power within the range of 150–300 W, and then decreased when ultrasonication power was above 300 W, mainly because the ultrasonication power exceeded the critical power value of cavitation; this effect led to the failure and collapse of cavitation bubbles [30]. The partial degradation of polysaccharides caused by excessive ultrasonication power may be another reason for the reduction in polysaccharide yield [31]. Therefore, ultrasonication powers of 250, 300, and 350 W were considered for the following tests.

The influence of liquid:solid ratio on AALP extraction rate is shown in Fig. 2(C). With the increase in liquid:solid ratio, AALP yield first increased and then slowly decreased, and a maximum AALP yield was obtained with a liquid:solid ratio of 20 mL/g. As the liquid:solid ratio increased, the mass concentration difference of polysaccharides between the solid and liquid phases increased. This effect led to the increase in mass transfer power and polysaccharide yield. However, the increase in solvent also resulted in the decrease in ultrasonication energy per unit volume, which reduced AALP yield [32]. The liquid:solid ratios of 15, 20, and 25 mL/g were selected for RSM tests.

The effect of extraction time within the range of 15–75 mins on AALP yield is illustrated in Fig. 2(D). AALP yield increased significantly with the extension of time to 15–50 mins and then decreased slowly after 50 mins. Although the prolongation of extraction time is conducive to the dissolution and diffusion of polysaccharides in solvents, long extraction times may cause polysaccharide degradation and affect polysaccharide yield [33]. In summary, extraction times of 30, 45, and 60 mins were chosen for response surface tests.

#### 3.1.2 Response surface optimization

The response surface design was established with four factors and three levels in accordance with the results of the single-factor test. The results are given in Table 2. The following fitted full-variable quadratic regression equation model was obtained on the basis of polynomial regression analysis by Design Expert 13: Y = 10.47 + 0.21A + 2383B + 0.1325C + 0.1608D − 0.2225AB − 0.025AC − 0.1825AD − 0.3525BC − 0.15BD + 0.065CD − 0.7982A^2^ − 0.8357B^2^ − 0.8695C^2^ − 0.517D^2^. The regression equation and variance analysis results of the partial regression coefficient are presented in Table 3.

**Table 2:** Scheme and results of Box·Behnken design.

Run	A: Temperature (^o^C)	B: Ultrasonic power (W)	C: Liquid-solid ratio (mL/g)	D: Ultrasonic Time (min)	AALP yield (%)
1	−1	1	0	0	9.11
2	0	−1	0	1	9.13
3	0	1	1	0	8.74
4	0	0	−1	−1	8.86
5	0	0	0	0	10.5
6	0	1	0	1	9.4
7	0	0	0	0	10.5
8	1	−1	0	0	9.03
9	0	1	−1	0	9.19
10	0	0	0	0	10.48
11	1	0	−1	0	8.94
12	−1	0	0	−1	8.59
13	1	1	0	0	9.05
14	0	−1	0	−1	8.53
15	−1	0	1	0	8.71
16	−1	−1	0	0	8.2
17	1	0	0	−1	9.36
18	0	−1	−1	0	8.09
19	0	−1	1	0	9.05
20	−1	0	−1	0	8.42
21	1	0	0	1	9.36
22	0	0	1	−1	9.03
23	0	0	0	0	10.37
24	0	0	−1	1	9.03
25	0	0	0	0	10.52
26	−1	0	0	1	9.32
27	0	0	1	1	9.46
28	0	1	0	−1	9.4
29	1	0	1	0	9.13

**Table 3:** ANOVA table for quadratic model.

Source	Sum of Squares	df	Mean Square	*F*-value	*P*-value	Significant levels
Model	12.73	14	0.9095	323.76	< 0.0001	**
A	0.5292	1	0.5292	188.38	< 0.0001	**
B	0.6816	1	0.6816	242.65	< 0.0001	**
C	0.2107	1	0.2107	75.00	< 0.0001	**
D	0.3104	1	0.3104	110.50	< 0.0001	**
AB	0.1980	1	0.1980	70.49	< 0.0001	**
AC	0.0025	1	0.0025	0.8899	0.3615	
AD	0.1332	1	0.1332	47.43	< 0.0001	**
BC	0.4970	1	0.4970	176.93	< 0.0001	**
BD	0.0900	1	0.0900	32.04	< 0.0001	**
CD	0.0169	1	0.0169	6.02	0.0279	*
A^2^	4.13	1	4.13	1471.33	< 0.0001	**
B^2^	4.53	1	4.53	1612.82	< 0.0001	**
C^2^	4.90	1	4.90	1745.71	< 0.0001	**
D^2^	1.73	1	1.73	617.18	< 0.0001	**
Residual	0.0393	14	0.0028			
Lack of Fit	0.0250	10	0.0025	0.6986	0.7068	
Pure Error	0.0143	4	0.0036			
Cor Total	12.77	28				

** indicates extremely significant difference (*P* < 0.01), * indicates significant difference (*P* < 0.05).

**Fig. 3:**
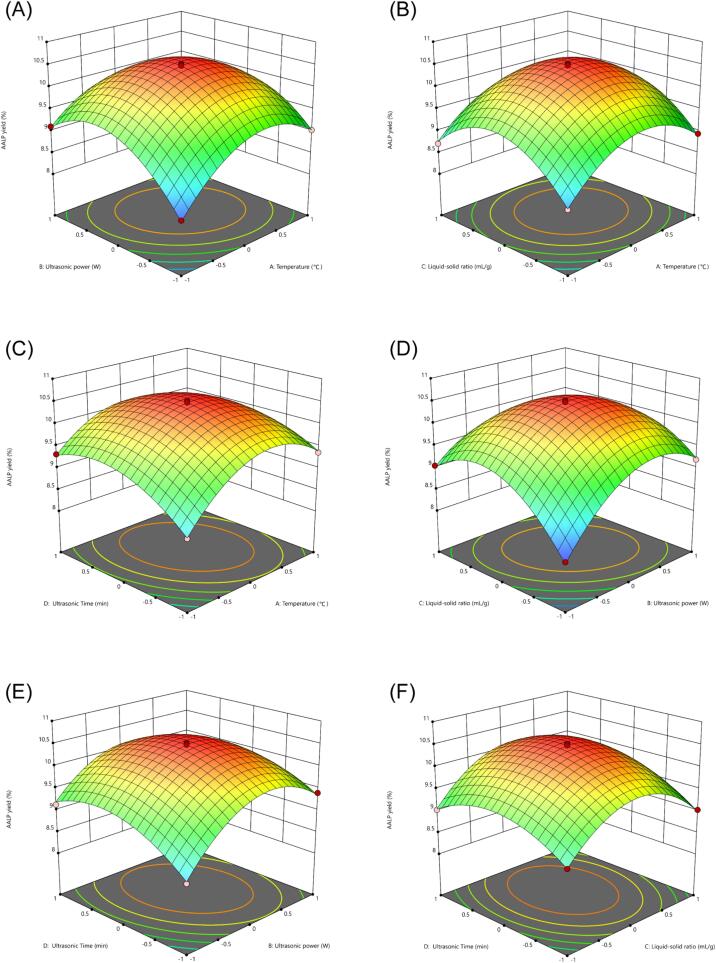
Response surface of two factor interactions on the yield of AALP. (a) AB 3D surface map. (b) AC 3D surface map. (c) AD 3D surface map. (d) BC 3D surface map. (e) BD 3D surface map (f) CD 3D surface map.

Table 3 shows that the regression model was highly significant (*p* < 0.001), and the lack of fit was insignificant due to a lack-of-fit *F* value of 0.7068. The model's predicted *R^2^* value of 0.9969 closely matched the adjusted *R^2^* value of 0.9938, indicating its suitability for analyzing and predicting AALP extraction. The small coefficient of variation (0.5746 %) suggested that the results were accurate and reliable. Adequate precision is a measure of signal-to-noise ratio, and a ratio above 4 indicates that a model has good anti-interference ability [34]. The adeq precision value of 63.7115 suggested that the model is accurate and reliable in the range of the independent variables. Furthermore, among all the four factors, ultrasonication power (B) acted as the most significant influential factor (*p* < 0.0001), followed by extraction temperature (A), time (D), and liquid:solid ratio (C). The interaction terms, except for AC (*p* > 0.05), were significant. As observed in Fig. 3, the response surfaces of AB, AD, BC, BD, and CD had a steep slope, showing that these interactions were highly significant, whereas AC was not. Among the four factors, temperature (A) and ultrasonication power (B) exerted the greatest influence on the yield of AALP. As these two factors increased, the AALP extraction rate also increased rapidly.

The optimal process conditions were identified through the analysis of the regression model, which included extraction temperature 51.01 °C, ultrasonication power 305.31 W, liquid:solid ratio 20.29 mL/g, and ultrasonication time 46.89 mins. Under these conditions, the predicted AALP extraction rate was 10.51 %. Extraction was performed in triplicate under the adjusted optimal conditions (51 °C, 300 W, 20 mL/g, and 47 mins) to validate the model’s reliability. The average AALP extraction rate was 10.49 % ± 0.06 %, which was closely consistent with the predicted value. Therefore, this model could be suitable for predicting extraction conditions.

### 3.2 Preliminary characterization of AALPs

#### 3.2.1 Chemical composition of AALPs

Given that AAL is a source of plant polysaccharides, examining the effect of HWE and UAE on its polysaccharides is crucial. Table 3 shows that the yield rate of AALP-H was 6.35 % ± 0.31 %, whereas that of AALP-U reached 10.49 % ± 0.52 %, indicating that UAE could significantly improve the extraction yield of AALPs (*p* < 0.05). Furthermore, compared with HWE, UAE had a shorter extraction time that supported considerably higher production efficiency. The yield of polysaccharides extracted from botanical sources is greatly influenced by the efficiency of cell wall and polysaccharide dissolution. Hot water caused slight cell wall damage, whereas UAE induced severe cell wall damage and hydrolysis, thereby enhancing polysaccharide dissolution. Consequently, UAE is a better choice than HWE for extracting AALPs.

The main chemical components of AALPs prepared through HWE and UAE are given in Table 4. The total sugar contents of AALP-H and AALP-U were 39.58 % ± 0.44 % and 48.65 % ± 0.52 % (*p* < 0.05), respectively, indicating that UAE could increase the total sugar content of AALPs because cavitation could accelerate cell wall destruction, thus helping to dissolve and release polysaccharides [35]. By contrast, due to its high temperature, HWE also promotes the dissolution of some nonpolysaccharide components, thus reducing the total sugar content [36]. In addition, the content of uronic acid in AALP-U was 18.93 % ± 0.60 %, which was considerably higher (*p* < 0.05) than that in AALP-H. This finding could be ascribed to the same reason for the total sugar content. Protein content showed the opposite trend as uronic acid. Moreover, the sulfate content in AALP-U was higher than that in AALP-H (*p* < 0.05). Peng *et al.* [37] reported that the negative charges carried by sulfate and uronic acid would considerably affect the functional properties of polysaccharides. In conclusion, the differences in extraction rates and chemical compositions of the resulting AALPs demonstrated that UAE was more efficient than HWE. Therefore, the functional activities of the AALPs were explored.

**Table 4:** Extraction yields and chemical compositions of AALP extracted by different methods.

	Yiled (%)	Total carbohydrates (%)	Uronic acids (%)	Proteins (%)	Sulfates (%)
AALP-U	10.49 ± 0.52^a^	48.65 ± 0.52^a^	18.93 ± 0.60^a^	2.52 ± 0.12^b^	8.70 ± 0.34^a^
AALP-H	6.35 ± 0.31^b^	39.58 ± 0.44^b^	10.07 ± 0.43^b^	6.18 ± 0.26^a^	6.40 ± 0.28^b^

Values indicated by different letters differ significantly (*P <* 0.05).

#### 3.2.2 Analysis of monosaccharide composition

**Fig. 4:**
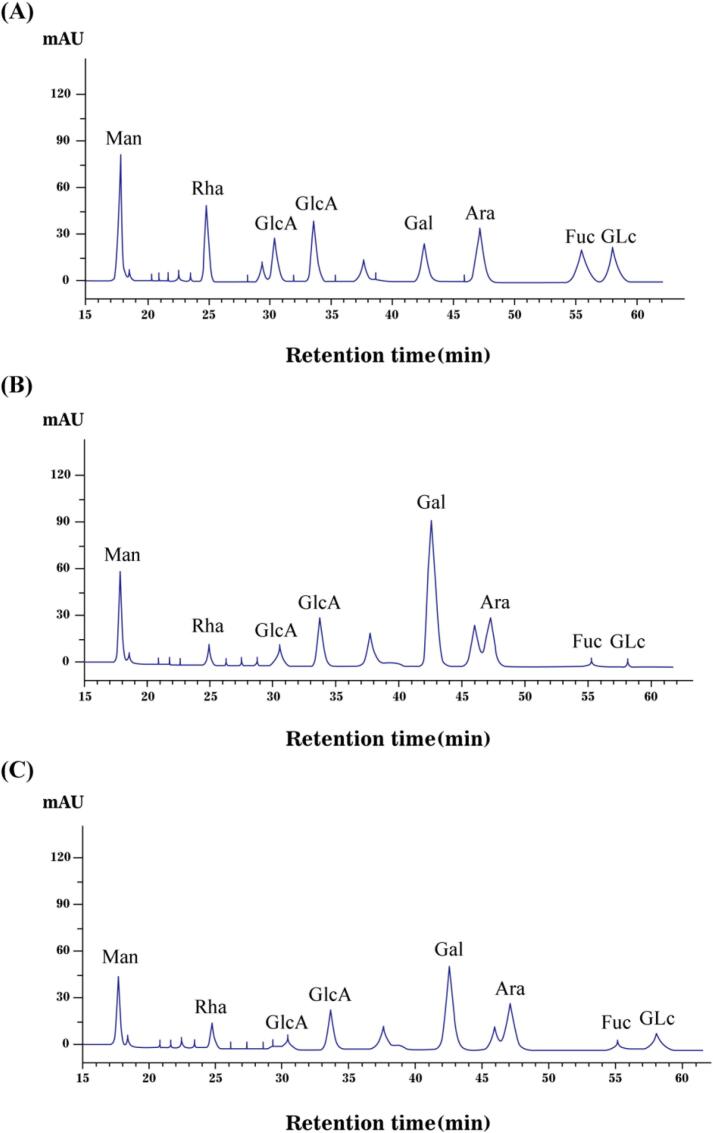
The monosaccharide mixed standard (A) and monosaccharide composition of AALP-U (B) and AALP-H (C) on HPLC instrument.

As shown in Fig. 4, monosaccharide composition analysis revealed that the AALPs prepared through two different extraction processes in this work were composed of eight types of monosaccharides: galactose, mannose, arabinose, galacturonic acid, GluA, rhamnose, fucose, and glucose. These findings were in line with that reported by Ruan *et al.* [38]. The main monosaccharide components of AALP-H and AALP-U were mannose, galactose, and arabinose, of which the total molar ratio exceeded 83 %, revealing that different extraction processes had little effect on monosaccharide composition but can remarkably affect the molar ratio of monosaccharides. Consistent with the results presented in 3.2.1, AALP-U had higher uronic acid content than AALP-H likely because UAE promoted polysaccharide chain hydrolysis and intermolecular hydrogen bond breakage, thus influencing the content of monosaccharides in polysaccharides [39].

#### 3.2.3 Determination of molecular weight

Table 5 presents the molecular weight distributions of AALP-U and AALP-H, which demonstrate that both polysaccharides contain four main components. In particular, the average molecular weight distribution of AALP-H was 1587.36 kDa (5.35 %), 451.26 kDa (6.96 %), 82.06 kDa (38.54 %) and 19.79 kDa (49.15 %), respectively. AALP-U also exhibited four distinct chromatographic peaks with molecular weights of 1868.45 kDa (4.63 %), 434.84 kDa (5.74 %), 83.07 kDa (53.17 %) and 12.68 kDa, respectively. The results demonstrate a significant disparity in the molecular weight distribution of polysaccharides obtained via HWE and UAE. This discrepancy may be attributed to the ultrasonic treatment's capacity to effectively alter the glycosidic bond and molecular weight distribution of polysaccharides, potentially leading to their degradation [40]. Additionally, under the influence of hot water, polysaccharides are prone to aggregation into macromolecules, resulting in an elevated average molecular weight due to the elevated extraction temperature and prolonged extraction duration [41].

**Table 5:** Molecular weight determination of AALP-U and AALP- H.

	AALP-U				AALP-H			
	Peak 1	Peak 2	Peak 3	Peak 4	Peak 1	Peak 2	Peak 3	Peak 4
RT (min)	8.06	10.84	11.85	13.39	8.12	10.86	11.94	13.41
MW (kDa)	1868.45	434.84	83.07	12.68	1857.36	451.26	82.06	19.79
Aera (%)	4.63	5.74	53.17	36.46	5.35	6.96	38.54	49.15

#### 3.2.4 Infrared spectroscopy

**Fig. 5:**
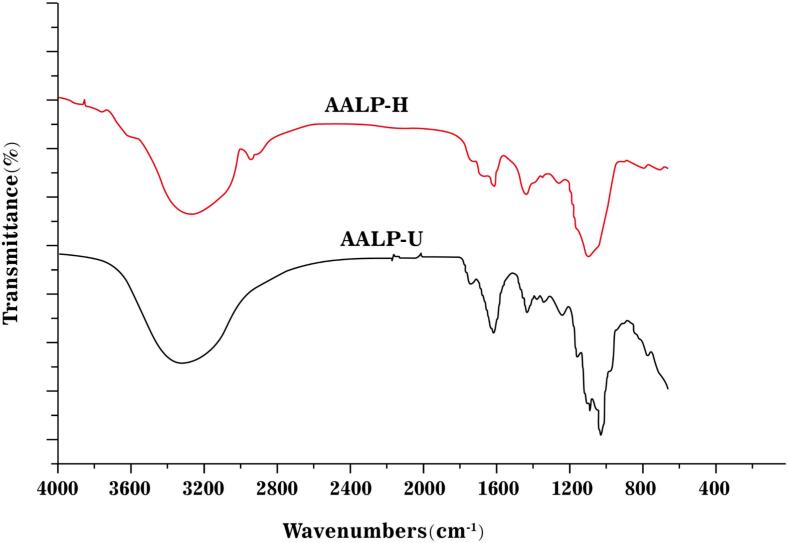
FT-IR spectra of AALP-H and AALP-U.

Fig. 5 shows that the typical bands of polysaccharides appeared at approximately 3275, 2930, 1615, 1420, and 1070 cm^−1^ in AALP-H and AALP-U, which had similar infrared absorption spectra. The infrared spectral bands were classified as follows: (1) The bands at 3275 cm^−1^ were caused by O–H stretching vibration [42]. (2) The band at 2930 and 1420 cm^−1^ was ascribed to C–H stretching vibration [43]. (3) The bands appearing in the range of 1600–1680 cm^−1^ (especially 1615 cm^−1^) were likely caused by thee asymmetric bending vibration of C = O, indicating that AALP-H and AALP-U are acidic polysaccharides [44]. In addition, the band of AALP-U at 1770 cm^−1^ was stronger than that of AALP-H, indicating that uronic acid was present and providing further proof that the content of uronic acid in AALP-U was higher than that in AALP-H [45]. (4) The band at 950–1200 cm^−1^ was assigned to the C–O–C stretching vibration in the pyranose ring [46]. (5) The band at 1230 cm^−1^ was the asymmetric stretching vibration of S–O, indicating that both polysaccharides contained sulfate [47]. In general, the polysaccharides prepared through HWE and UAE were not significantly different.

#### 3.2.5 Particle sizes and zeta potentials of AALPs

The particle sizes and zeta potentials of polysaccharides are related to stability. Therefore, the fluidity and solubility of polysaccharides in solution were identified. Generally speaking, small particle sizes and low absolute zeta potential values indicate that polysaccharide molecules are easily dispersed in the dissolution system and easily aggregate and exhibit low solubility otherwise [48]. Table 6 shows that the average particle sizes of AALP-H and AALP-U were 237.50 ± 17.39 and 149.80 ± 8.33 nm, respectively, indicating that UAE could considerably reduce the average AALP particle size (*p* < 0.05). Furthermore, the zeta potential value of AALP-U was |−16.43 ± 0.65| mV and higher than that of AALP-H (|−14.52 ± 0.56|mV) (*p* < 0.05). The above chemical composition analysis revealed that uronic acid and sulfate mainly contributed to the negative charges of AALPs and zeta potential value was closely aligned with the total contents of uronic acid and sulfate in AALP-H and AALP-U. Therefore, the small average particle size and high absolute zeta potential value of AALP-U supported its good stability.

**Table 6:** Particle sizes and Zeta potentials of AALP-H and AALP-U.

	Particle sizes (nm)	Zeta potentials (mV)
AALP-H	237.50 ± 17.39^a^	−14.52 ± 0.56^a^
AALP-U	149.80 ± 8.33^b^	−16.43 ± 0.65^b^

Values indicated by different letters differ significantly (*P <* 0.05).

### 3.3 In vitro antioxidant activity of AALPs

**Fig. 6:**
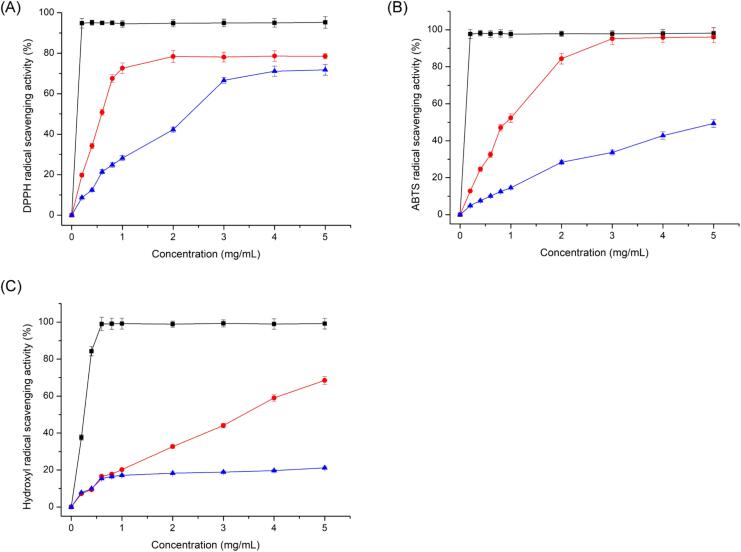
Antioxidant activities of AALPs in vitro. DPPH radical scavenging activity (A), ABTS radical scavenging activity (B), hydroxyl radical scavenging activity (C). Ascorbic acid was the positive control represented by black line, AALP-U by red line and AALP-H by blue line.

DPPH is a class of extremely stable free radicals that can remain undecomposed almost indefinitely, and previous studies have proven that DPPH free radicals play an important role in the evaluation of the antioxidant activity of natural extracts [49]. As illustrated in Fig. 6 (A), both AALPs exhibited dose-dependent scavenging effects on DPPH free radicals, and the sample concentration of 5 mg/mL provided a maximal scavenging rate of 78.5 % for AALP-U, which was considerably higher than that for AALP-H.

ABTS free radicals have been extensively used to determine total antioxidant activity [50]. Fig. 6 (B) illustrates that both polysaccharides have the ability to scavenge ABTS free radicals. However, the scavenging activity of AALP-U was remarkably stronger than that of AALP-H. When its concentration exceeded 3.0 mg/mL, the scavenging rate of AALP-U could approach 100 %, which reached that of the positive control ascorbic acid.

In organisms, hydroxyl free radicals can attack various macromolecules, such as carbohydrates, lipids, amino acids, and nucleic acids, and are deemed to be the most harmful free radicals among ROS [51]. Therefore, protecting body cells or food systems from oxidative damage by removing hydroxyl free radicals is critical. Fig. 6 (C) illustrates that the scavenging abilities of AALP-H and AALP-U for hydroxyl free radicals were lower than that of the positive control ascorbic acid. At the concentration of 5 mg/mL, the scavenging rates for hydroxyl radicals followed the order of ascorbic acid > AALP-U > AALP-H. These results suggested that AALPs could be utilized as a potential antioxidant that provides electrons or hydrogen to scavenge hydroxyl radicals.

The results of the in vitro antioxidant activity study demonstrated that although the polysaccharides obtained through HWE and UAE had significant scavenging ability for ABTS, DPPH, and OH free radicals, the in vitro antioxidant activity of AALP-U was stronger than that of AALP-H. This finding further proved that different extraction processes could greatly affect the antioxidant activity of AALPs in vitro. Compared with AALP-H, AALP-U had higher total sugar, uronic acid, and sulfate contents (Table 4) that may be conducive to enhancing its electron or hydrogen atom supply capacity, thereby improving its free radical scavenging capacity [52]. Therefore, AALP-U was selected for the subsequent preliminary evaluation of antifatigue activity.

### 3.4 Preliminary exploration of the antifatigue activity of AALPs

#### 3.4.1 Effects of AALPs on the weight-bearing swimming time of mice

**Fig. 7:**
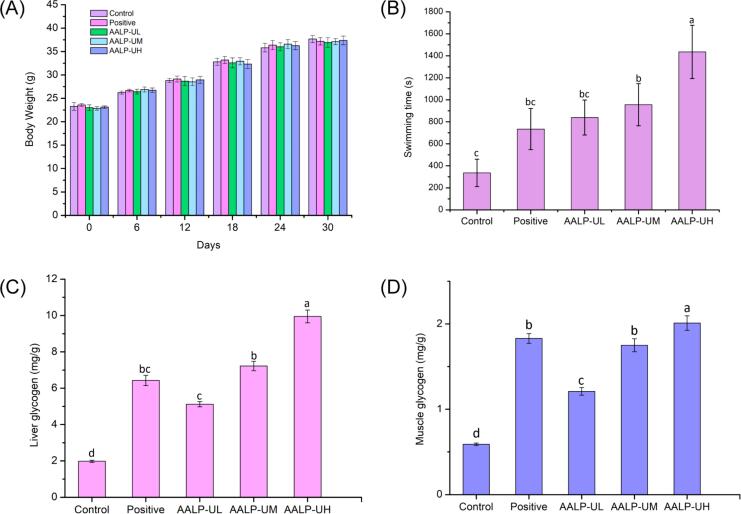
The effect of AALPs on body weight (A), weight-bearing swimming time (B), liver glycogen (C) and muscle glycogen (D). The different letters represent significant differences (*p* < 0.05).

Fatigue leads to changes in exercise ability. Exercise time extends as the body's antifatigue ability strengthens [53]. The weight-bearing swimming experiment is a recognized model that can effectively reflect exercise endurance and has been widely used to assess the antifatigue activity of various bioactive substances [54]. The effects of AALP-U on the body weight and weight-bearing swimming time of mice are shown in Fig. 7 (A, B). After 30 days of gavage treatment, the mice in all groups had stable vital signs and no incidences of death and exhibited increased weight without significant differences among the five groups (*p* < 0.05). This finding indicated that the experimental dose lacked toxicity. Compared with that in the control group (336 ± 84.6 s), the exhaustive swimming times of the mice in the low-, middle-, and high dose groups of AALP-U increased to 473 ± 88, 689 ± 99, 1065 ± 172 s, respectively, whereas that of the mice in the positive control group was 841 ± 139 s. The middle and high doses of AALP-U could significantly prolong the swimming time of weight-bearing mice (*p* < 0.05) in a dose-dependent manner. In summary, in weight-bearing mice, AALP-U could prolong swimming time and improve exercise endurance.

#### 3.4.2 Effects of AALPs on glycogen levels in mice

Glycogen is a major source of body energy and plays an important role in prolonging the body's exercise time. Muscle glycogen is the major form of glucose reserves in the human body. It can induce muscle movement and rapidly provide energy for muscle contraction. Long-term, high-intensity exercise consumes considerable amounts of muscle glycogen and affects the body's exercise ability [55]. When liver glycogen is exhausted during long-term exercise, maintaining normal levels of blood glucose becomes difficult, and the resulting hypoglycemia may lead to neurological impairment. Therefore, increasing liver glycogen levels is conducive to the body's maintenance of blood glucose levels during intense exercise, thus enhancing exercise endurance [56]. High-intensity exercise leads to massive glycogen consumption, which causes hypoglycemia, fatigue, and other uncomfortable symptoms. Therefore, muscle and liver glycogen levels are biochemical parameters for determining body fatigue, and their increase can verify the antifatigue efficacy of tested substances [57]. Fig. 7 (C, D) show that the in mice, the average muscle and liver glycogen levels decreased significantly after 30 mins of swimming (*p* < 0.05), revealing that the mice were in an extremely fatigued state after swimming. The liver and muscle glycogen contents in all treatment groups (AALP-UL, AALP-UM, and AALP-UH) were significantly higher than those in the control group (*p* < 0.05), demonstrating that AALP-U functioned to increase glycogen reserves, accelerate energy supply, relieve fatigue, and enhance exercise endurance. Moreover, liver and muscle glycogen contents were higher in the high-dose group than in the medium- and low-dose groups, indicating dose dependence. Therefore, AALPs can up-regulate the level of glycogen in the body and regulate energy metabolism during exercise to play an antifatigue role.

#### 3.4.3 Effects of AALPs on oxidative stress

**Fig. 8:**
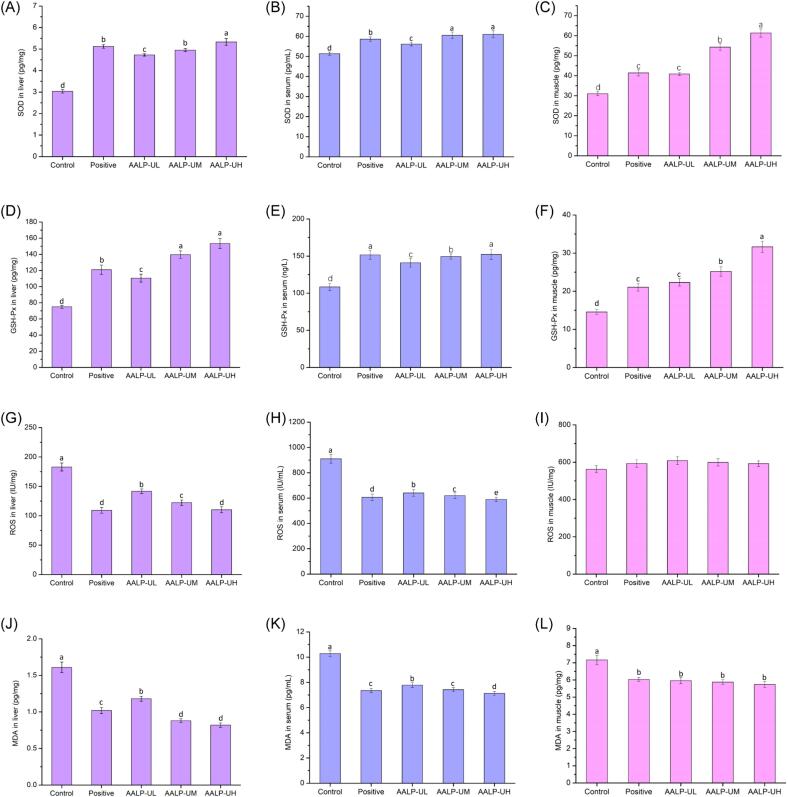
The effect of AALPs on oxidative stress (A) ROS in liver (B) ROS in serum (C) ROS in muscle, (D) SOD in liver (E) SOD in serum (F) SOD in muscle, (G) MDA in liver (H) MDA in serum (I) MDA in muscle, (J) GSH-Px in liver (K) GSH-Px in serum (L) GSH-Px in muscle. The different letters represent significant differences (*p* < 0.05).

Free radicals are highly reactive chemicals with unpaired electrons and are therefore highly unstable. When free radicals accumulate in excess, they can cause damage to human cells. Such damage is a principal factor leading to oxidative stress and muscle fatigue. However, free radicals can be cleared by antioxidant enzymes, such as SOD and GSH-Px. Therefore, antioxidants can prevent or reduce oxidative stress effectively and further improve exercise performance. Serum, liver, and muscle SOD and GSH-Px contents in mice in each group were measured (Fig. 8[A–F]). The results implied that compared with the control treatment, AALP-U significantly increased the levels of SOD and GSH-Px in serum, liver, and muscle (*p* < 0.05). This effect showed a certain dose dependence.

Excessive exercise leads to a sharp increase in ROS and severe oxidative stress, which constitutes another cause of fatigue [58], [59]. ROS contents in the liver, muscle, and serum of mice were measured, and the results are presented in Fig. 8(G–I). Compared with those in the control group, the ROS levels in the liver and serum of the mice in the ginsenoside and all AALP-U groups decreased significantly after treatment (*p* < 0.05). This finding preliminarily proved that AALP-U could effectively reduce ROS levels in the liver and serum of mice with acute fatigue.

MDA, a key index of the body's potential antioxidant capacity, mainly mirrors the rate of lipid peroxidation in the body and further reflects the degree of cell membrane damage. High MDA contents in the body are indicative of severe cell damage. Fig. 8(J–L) illustrate that compared with the control treatment, ginsenoside and AALP-U treatment significantly reduced MDA levels in the liver and serum (*p* < 0.05). This effect showed a certain dose dependence, indicating that AALP-U could protect the body from fatigue-induced oxidative damage. In conclusion, in mice, AALP-U can dramatically increase SOD and GSH-Px activities and reduce ROS and MDA accumulation. AALP-U not only participates in antifatigue activities, it also acts as a beneficial antioxidant. Therefore, its resistance to oxidative stress may be one of its antifatigue mechanisms.

However, it should be noted that this research has two limitations. Firstly, all the evaluation results were achieved by analysis based on the crude AALP-H and AALP-U, which are not purified. Besides, the future study should concentrate on verification experiments which help to explain the evaluation results of this study.

## 4 Conclusion

AAL was used to prepare polysaccharides through UAE. Extraction was optimized by RSM. The optimal extraction conditions were ultrasonic power 300 W, extraction temperature 51 °C, liquid:solid ratio 20 mL/g and ultrasonic time 47 mins. The above conditions provided a maximal extraction rate of 10.49 %, which was consistent with the theoretical predicted value, hence confirming the reliability of the model. AALP-U showed higher stability and stronger antioxidant activity than AALP-H and was therefore selected for the antifatigue activity test. Compared with the positive treatment, AALP-U at all doses significantly prolonged the exhaustion swimming time, increased glycogen reserves, and improved biochemical parameters. In summary, AALP-U exhibited considerable antifatigue activity and has excellent application prospects in the field of food and medicine.

## CRediT authorship contribution statement

**Jiahui Yu:** Data curation. **Ying Cao:** Formal analysis. **Feng He:** Investigation. **Fu Xiang:** Resources. **Shuzhen Wang:** Validation. **Wenbing Ke:** Writing – review & editing. **Wei Wu:** Writing – original draft, Supervision, Funding acquisition, Conceptualization.

## Declaration of competing interest

The authors declare that they have no known competing financial interests or personal relationships that could have appeared to influence the work reported in this paper.
