# Supplementary figures and images for: Polysaccharides from Artemisia argyi leaves: Environmentally friendly ultrasound-assisted extraction and antifatigue activities
Source: Ultrason Sonochem. 2024 May 31;107:106932. doi: 10.1016/j.ultsonch.2024.106932 (PMC11170280; doi:10.1016/j.ultsonch.2024.106932)

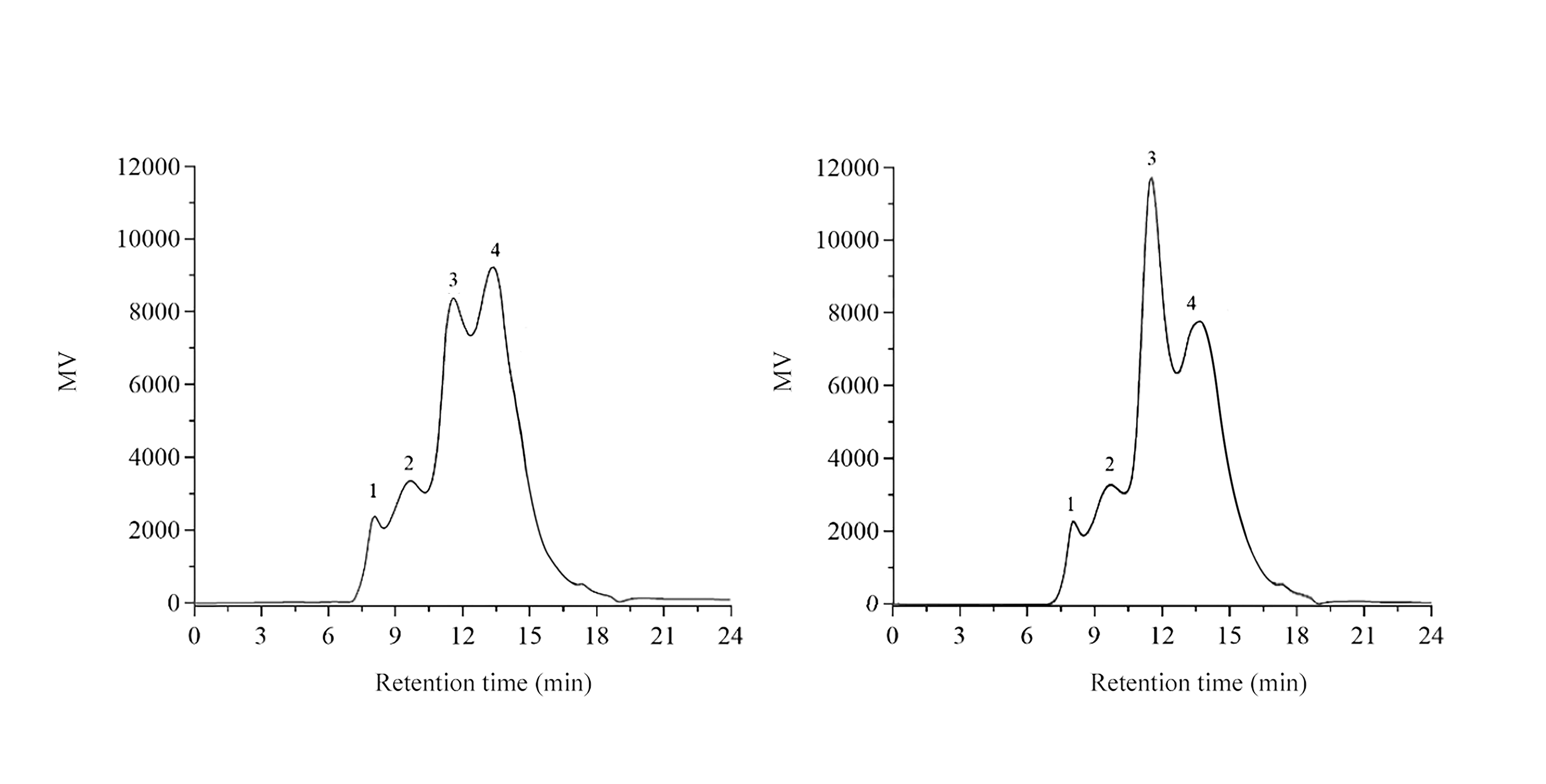


A

B

Fig.1 The HPGPC chromatograms of AALP-HWE (A) and AALP-U(B)

Supplement: Supplementary Data 1 [file mmc1.docx]
